# Supplementary material for: Outcomes of Pediatric Liver Transplantation in Korea Using Two National Registries
Source: J Clin Med. 2020 Oct 26;9(11):3435. doi: 10.3390/jcm9113435 (PMC7694033; doi:10.3390/jcm9113435)
Supplement: Supplementary file 1 [file jcm-09-03435-s001.pdf]

Supplementary material

Outcomes of pediatric liver transplantation in Korea using two national registries

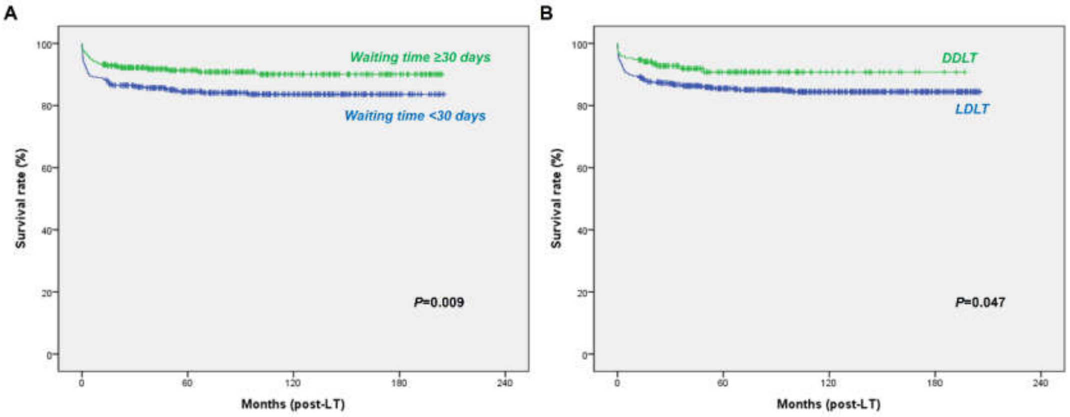

Figure S1. Kaplan-Meier analysis of overall survival using the KONOS database according to (**A**) waiting time, (**B**) type of LT.

Table S1. Initial postoperative immunosuppression.

Immunosuppresion	Number of patients (%)
Tacrolimus+basiliximab+steroid	35 (46.1)
Tacrolimus+steroid	21 (27.6)
Tacrolimus+basiliximab+MMF+steroid	11 (14.5)
Tacrolimus+basiliximab+MMF	2 (2.6)
Tacrolimus only	1 (1.3)
Tacrolimus+basiliximab	1 (1.3)
Tacrolimus+MMF	1 (1.3)
Tacrolimus+MMF+steroid	1 (1.3)
Basiliximab+steroid	1 (1.3)
Steroid only	1 (1.3)
None	1 (1.3)

mycophenolate mofetil, MMF
